# Heart Rate Variability Analysis in General Medicine

**Published:** 2003-01-01

**Authors:** Yi Gang, Marek Malik

**Affiliations:** St. George's Hospital Medical School, London, UNITED KINDOM

**Keywords:** heart rate variability, autonomic modulation, risk assessment

## Abstract

Autonomic nervous system plays an integral role in homeostasis. Autonomic modulation can frequently be altered in patients with cardiac disorders as well as in patients with other critical illnesses or injuries. Assessment of heart rate variability is based on analysis of consecutive normal R-R intervals and may provide quantitative information on the modulation of cardiac vagal and sympathetic nerve input. The hypothesis that depressed heart rate variability may occur over a broad range of illness and injury, and may inversely correlated with disease severity and outcome has been tested in various clinical settings over the last decade. This article reviews recent literature concerning the potential clinical implications and limitations of heart rate variability assessment in general medicine.

Autonomic nervous system plays an integral role in many aspects of homeostasis, and it is affected by a wide variety of pathophysiologic conditions. Assessment of heart rate variability (HRV) is based on the analysis of consecutive sinus rhythm R-R intervals and may provide quantitative information about the modulation of cardiac vagal and sympathetic nerve activities. HRV measurements can be derived from short-term (2 to 5 minutes) or long-term ECG recordings (24 to 48 hours). It can be quantified in a number of ways but techniques of conventional time domain (statistical and geometrical) and frequency domain measurements (power spectral density) remain predominantly utilised [1]. Recently, analysis of heart rate dynamics by methods based on non-linear system theory has been introduced, which may be an alternative way for studying the abnormalities in heart rate behaviour and for identifying patients with an increased cardiac mortality [2,3]. Although HRV has been used as a non-invasive marker of the activity of the autonomic nervous system for over two decades, the necessary guidelines have only been established rather recently making comparison feasible between various studies [1].

 Autonomic nervous system plays a central role in the maintenance of hemodynamic stability. Cardiac autonomic dysfunction may result in serious complications, such as malignant cardiac arrhythmias and/or sudden cardiac death. The analysis of HRV has been widely explored in cardiac research during last decades. HRV has been recognised as a powerful risk stratifier for adverse cardiac events in patients surviving myocardial infarction and for identifying patients with diabetic neuropathy. HRV is affected by a number of physiological and pathological conditions. Significantly altered HRV can be found not only in cardiac diseases but also in a wide variety of pathophysiologic disorders characterised by neurohumoral activation [4].

 Physiologic systems constantly change over time and respond to stimuli. Physiologic systems of young healthy subjects exhibit marked physiological signal variability and complexity, while aging or diseased systems show a loss of variability, decreased complexity, and increased regularity [5]. The loss or decreased variability in the beat-to-beat intervals has been found in various pathophysiologic disorders [4-9]. It has been hypothesised that decreased variability of heart rate dynamics may occur over a broad range of critical illness and injury, and may be inversely correlated with disease severity and outcome in both adult and paediatric patients. These hypotheses have been tested in last decade and have continuously been evaluated in the areas of both cardiac and general medicine.

## Clinical application of HRV analysis

Autonomic dysfunction is frequently seen in cardiac disorders or other diseases including those requiring admission to intensive care unit such as myocardial infarction [7,8], multiple organ dysfunction syndrome, sepsis [10], and severe head and brain injuries [11]. Early studies have clearly showed that HRV is significantly depressed and associated with prognosis in post myocardial infarction patients [1]. Recently, there have been reports suggesting that HRV is significantly altered in patients with critical illness, and associated with severity of illness and outcome in patients with head injury or brain death [6,12-18]. The phenomenon of decomplexification of physiologic dynamics measured by HRV may also have important clinical implications in other critically ill patients [16,19-21].

### Cardiovascular risk stratification

#### Myocardial infarction and angina pectoris

A general consensus of a practical use of HRV has been reached in the setting of myocardial infarction. Depressed HRV indicates increased risk of malignant arrhythmias and of mortality after acute myocardial infarction and the prognostic value of time and geometric parameters of HRV has been consistently confirmed [1]. Carney et al showed by analysis of covariance that HRV index, with the exception of the high-frequency components, remains significantly lower in patients with a recent myocardial infarction and depression compared to those without depression. This finding may suggest a mechanism linking depression to increased cardiac mortality in post infarction patients [7]. More recently, a prospective multicentre study showed that [1], a fractal HRV parameters, was the most significant independent HRV index which predicted subsequent mortality in a multivariate analysis after adjustments for clinical variables and left ventricular ejection fraction (relative risk 3.90, 95% CI 2.03 to 7.49, p <0.001) based on 697 survivors of acute myocardial infarction [9]. Forslund et al. reported that in patients with stable angina pectoris, low HRV predicted cardiovascular death but not non-fatal myocardial infarction [22].

#### Congestive heart failure

A number of recent studies confirmed that HRV was significantly depressed in patients with decompensated heart failure and assessment of HRV could be used to identify heart failure patients with poor prognosis [8,23-25]. Boveda et al examined the time domain analysis of HRV in 190 patients with heart failure and found that depressed HRV was of independent prognostic value [23]. A multicentre trial (n=1071) ATRAMI demonstrated that HRV, along with baroreflex sensitivity, contributed importantly and additionally to risk stratification in post-infarction patients and with left ventricular ejection fraction <35% [8]. Carvedilol, spironolactone and beta-blockers therapy reduced heart rate and improved HRV in congestive heart failure [26-29]. These effects may be attributed to the improvement of autonomic dysfunction [27]. Differently, Makikallio et al found that HRV indexes, including the standard deviation of RR intervals, HRV index, frequency-domain indexes, and the short-term fractal scaling exponent of RR intervals, failed to provide independent prognostic information in patients with the most severe functional impairment [30]. More recently, Bilchick et al. reported that patients with SDNN <65.3 ms had a significantly increased risk of sudden death (p = 0.016). They showed that HRV was the sole independent predictor of overall mortality and was significantly associated with sudden death in patients with ischaemic cardiomyopathy [24].

#### Malignant arrhythmias and sudden cardiac death

More recent report from ATRAMI confirmed the predictive value of HRV for life-threatening arrhythmias in patients after myocardial infarction [8]. HRV (SDNN, SDANN) was also found to be significantly reduced in sudden death survivors of various aetiology [31]. Schmitt et al [32] performed a two-step risk stratification to identify patients who were at high risk of ventricular arrhythmias after acute myocardial infarction, and used HRV as one of the non-invasive screening tests in 1,436 consecutive post-infarction patients. A subgroup of 248 patients was identified at high-risk and scheduled for programmed ventricular stimulation. During a mean of 607 day follow-up, cardiac mortality was significantly higher in the subgroup of 96 high-risk patients who declined electrophysiological study (log-rank chi-square 9.38, p = 0.0022, relative risk 4.7, 1.6 to 13.9) compared to the high-risk group of patients (n=98) in whom a subsequent ICD implantation was guided by electrophysiological testing [32]. In patients with implantable cardioverter-defibrillators, Perkiomaki et al found that non-linear measure of HRV (scaling exponent 1) obtained from short-term ECG recordings yielded important prognostic information about the risk of appropriate ICD shocks and/or death [33].

#### Essential hypertension

It has been hypothesised that in essential hypertension, an increased sympathetic and reduced vagal cardiac drive is coupled with an enhancement of vasomotor sympathetic modulation [34]. Essential hypertension was associated with impaired cardiac autonomic function [35,36]. In a recent study, Mussalo et al showed that the severity of essential hypertension was related to the severity of impairment of cardiac autonomic control measured by time and frequency domain analysis of HRV [37].

#### Cardiovascular surgery

Both conventional and novel (non-linear) analysis of HRV have been applied to evaluate autonomic modulation and cardiovascular function in patients after cardiovascular surgery [38-40]. Stein et al [40] examined the value of HRV measurements in predicting clinical course in 106 patients undergoing abdominal aortic surgery. 24-hour Holter recordings taken on postoperative day 1 were analysed for standard time and frequency domain indices as well as non-linear slope of HRV. Decreased HRV along with increased age and insulin-dependent diabetes were shown to be independent predictors of prolonged hospitalisation (>7 days) after the operation. Their findings suggest the potential use of HRV for the prediction of postsurgical resource utilisation. HRV analysis was also applied to the assessment of the neural circulatory control after cardiac transplantation [39]. Altered HRV was associated with myocardial ischaemic episodes in patients after CABG [41]. Few data exist regarding the value of preoperative HRV analysis for risk assessment before vascular surgery [42].

### Assessment of risk in neurologic disorders

#### Severe head injury and brain death

About two decades ago, Lowensohn tested whether or not the characteristics of the adult heart rate reflected the condition of the central nervous system as they appear to characterise it in the fetus. They found that the normal cyclic changes in heart rate were reduced in the presence of severe brain damage. HRV decreased rapidly when intracranial pressure rose, and the rate of return of variability reflected the subsequent state of neuronal function, even when intracranial pressure was restored to normal. Thus, it appears in this limited setting that HRV may reflect the functional state of the central nervous system [17]. Power spectral analysis of HRV was later proposed as a tool to diagnose and monitor brain damage [11,14]. In 1990s Goldstein et al demonstrated a strong negative correlation between HRV (low frequency power) and severity of brain injury, survival, and neurologic outcome in paediatric populations [14-16]. More recently, Conci et al evaluated spontaneous variability of heart rate estimated by pulse interval in 11 patients shortly before and 1 hour after the onset of brain death. Significant spectral changes occurred after brain death with a general power reduction in pulse interval spectra; A significant shift of pulse interval powers toward the lower frequencies occurred after brain death together with a general power reduction in pulse interval spectra parameters describing spontaneous HRV underwent marked changes with the onset of brain death. Most likely, the changes reflect the cessation of activity of the cardiovascular brain stem centres. Hence, Conci et al suggested that spectral analysis of HRV, along with the analysis of baroreflex sensitivity and blood pressure variability, may be used as a complementary tool to confirm the diagnosis of brain stem death [13]. Rapenne et al prospectively investigated whether HRV analysis could predict outcome in patients with severe head injury (n=20). They compared HRV (on Day 1) in patients who progressed to brain death to HRV in survivors; as well as HRV in survivors with good recovery (Glasgow Coma Scale >or= 10) to HRV in those with worsened neurologic state (Glasgow Coma Scale < 10). At day 1, in the 6 patients who had progressed to brain death, global HRV and parasympathetic tone were significantly higher, while during the awaking period, global HRV and the parasympathetic tone were significantly lower in the worsened neurologic state group. These findings suggest that HRV may be a helpful predictor of imminent brain death and a useful adjunct for predicting the outcome of patients with severe head injury [43]. Baillard et al [6] further evaluated HRV continuously during the time of brain death. They showed that an improved method of HRV analysis immediately identified the loss in the spectral power of heart rate occurring during transition to brain death. This approach may be used to confirm the cessation of brainstem function in clinical practice.

#### Acute brainstem stroke

 Meglic et al assessed HRV by power spectra analysis in 14 patients with acute brainstem stroke. Transient dysfunction of autonomic nervous system was found in patients with acute medullary stroke (n=6) in contrast to those with non-medullary (n=8) brainstem stroke [44]. However, the size of the study population may limit the value of this report.

#### Guillain-Barre syndrome

Flachenecker et al conducted serial studies in patients with Guillain-Barre syndrome and found that the sympathovagal balance shifted to sympathetic predominance at the height of the disease [45-47]. The authors concluded that the 24-hour heart rate power spectrum may yield sensitive and specific markers for assessing the risk of impending and potentially life-threatening arrhythmias in patients with Guillain-Barre syndrome.

### Risk assessment in renal failure

Cardiac autonomic dysfunction is associated with mortality in patients with end-stage renal disease. Cashion et al evaluated the value of HRV analysis in 278 patients with end-stage renal disease to identify those at high risk of sudden cardiac death. They showed that end-stage renal disease patients, particularly diabetics, had a compromised autonomic function. Although they suggest that 24-hour time domain measure of the HRV held the promise of identifying patients at increased risk of death, this study contained only 5 patients with sudden cardiac death [48]. In patients with end-stage renal disease who underwent chronic haemodialysis, uraemia causes similar but reversible changes in HRV in non-diabetic haemodialysis patients [49].

### Evaluation outcomes in sepsis and shock

Godin et al prospectively studied the relation between human endotoxemia to loss of the physiologic beat-to-beat variability of heart rate. They found that endotoxin administration was associated with loss of the variability by all measures of HRV in healthy volunteers [50]. Later findings obtained from patients were in agreement with the experimental results from healthy subjects. Korach et al found that sepsis was strongly associated with a low to high frequency ration <1.50, with an odds ratio of 3.63 (95% confidence interval 1.47 - 9.01, p=0.005) in a study of 41 patients. The authors suggested that a low to high frequency ratio <1.0 may be a diagnostic test for sepsis [10]. However, the reproducibility of the low to high frequency ratio is a reason for concern and these findings need to be confirmed by studying larger populations.

It was hypothesised that septic shock resulted in an uncoupling of organ system interconnectivity and that the uncoupling phenomenon could be quantified as a loss in HRV [51]. Goldstein et al showed that endotoxin caused a loss in HRV in rabbit models of sepsis. They speculated that a concomitant decrease in low-frequency HR power as mean arterial pressure decreases may be an early marker for impending shock [52]. Ellenby et al evaluated uncoupling and recoupling phenomenon in 7 children with septic shock by observing serial changes in HRV. Heart rate time series were analysed at 6-hour intervals of during hospitalisation at paediatric intensive care unit. Six of 7 patients showed an increase over time in low-frequency component of HRV and the low to high-frequency ratio, whereas high-frequency component of HRV decreased. The study also compared the change in mean heart rate, heart rate standard deviation, and power spectral HRV values during the first 24-hour of paediatric intensive care unit hospitalisation versus the remainder of the stay. In the later hospitalisation, low-frequency component of HRV and the low-/high-frequency ratio increased, whereas high-frequency component of HRV decreased over the course of the illness. This report showed the potential value of monitoring HRV in paediatric patients with septic shock [51]. These results were in agreement with early studies showing evidence of decomplexification in experimental models of sepsis [52] and with recent clinical studies [10,53].

### Fetal and neonatal monitoring

As early as in 1965, Hon and Lee noted that fetal distress was preceded by alterations in the beat-to-beat intervals before any appreciable changes occurred in heart rate itself [54]. More recently, Griffin et al prospectively evaluated the heart rate characteristics, which described the symmetry of histogram of RR intervals, in 89 infants at risk for sepsis and sepsis-like illness. They found that new-born infants who had abrupt clinical deterioration as a result of sepsis and sepsis-like illness, had abnormal heart rate characteristics. Heart rate characteristics and the Score for Neonatal Acute Physiology worsened over 24 hours before the clinical suspicion of sepsis. Their findings suggested that HRV analysis might help with an earlier diagnosis and more effective therapy [55]. Chung et al performed a fetal HRV analysis in 76 pregnant women. They reported that a computerised spectral analysis of fetal HRV was a good predictor of fetal distress by showing a negative predictive accuracy for predicting presumed distress or acidemic distress of 99%. However, the positive predictive accuracy was only 12% [56]. The effect of maternal administration of meperidine with promethazine should be taken into account when monitoring fetal HRV during the active phase of normal labour [57].

### Other clinical potential

A number of recent studies showed that altered HRV was found in other patients populations including Parkinson disease [58], sleep apnoea [59,60], Chagas' disease [61] etc. Altered HRV was also associated with some critical conditions such as patients with cirrhosis of the liver [62], pregnant eclampsia [63], and tetanus [64]. However, the clinical use of HRV assessment in these areas has not been systematically explored.

### Future prospects

The expansion of heart rate variability analysis has been facilitated by the remarkable development of computer sciences and digital signal processing during the last decades. The prognostic value of time and geometric parameters of HRV has been consistently confirmed. It seems encouraging that HRV analysis may provide additional diagnostic and prognostic information within the context of multiple confounding factors, which are associated with various pathophysiologic conditions. It has been known that both linear and non-linear components of HRV may be significantly altered in critically ill patients [65]. The newer non-linear methods might predict risk more precisely in cardiac or other critically ill patients [2,9,66], however, further evaluations are needed. This non-invasive methodology is of substantial utility to evaluate autonomic control mechanisms and to identify patients with an increased mortality in spite of an incomplete understanding of the physiological significance of HRV parameters. Further prospective studies are required to define limits of HRV indices for the use in managing individual patient under various clinical settings.

## Conclusions

HRV analysis is a recognised tool for the estimation of cardiac autonomic modulations. Reduced HRV is a powerful and independent predictor of an adverse prognosis in patients with cardiac disease. It has a potential to become a non-invasive diagnostic and prognostic index in clinical practice, particularly in patients with post myocardial infarction and diabetic neuropathy. Although assessment of HRV in other clinical settings seems to be helpful and promising, substantial number of further prospective studies in larger population are needed to evaluate the validity of HRV in general medicine.
